# Can selenium levels act as a marker of colorectal cancer risk?

**DOI:** 10.1186/1471-2407-13-214

**Published:** 2013-04-29

**Authors:** Marcin R Lener, Satish Gupta, Rodney J Scott, Martin Tootsi, Maria Kulp, Mari-Liis Tammesoo, Anu Viitak, Anders Metspalu, Pablo Serrano-Fernández, Józef Kładny, Katarzyna Jaworska-Bieniek, Katarzyna Durda, Magdalena Muszyńska, Grzegorz Sukiennicki, Anna Jakubowska, Jan Lubiński

**Affiliations:** 1 Department of Genetics and Pathology, International Hereditary Cancer Center, Pomeranian Medical University, Połabska 4, 70-115, Szczecin, Poland; 2 Postgraduate School of Molecular Medicine Medical University of Warsaw, Żwirki i Wigury 61, 02-091, Warsaw, Poland; 3 Discipline of Medical Genetics, School of Biomedical Sciences, Faculty of Health, University of Newcastle, Newcastle, NSW 2308, Australia; 4 The Hunter Medical Research Institute, Newcastle, NSW 2308, Australia; 5 The Estonian Genome Center, University of Tartu, 23b Riia Str., Tartu 51010, Estonia; 6 Tallinn University of Technology, Department of Analytical Chemistry, Akadeemia tee 1512618, Tallinn, Estonia; 7 Department of General and Oncological Surgery, Pomeranian Medical University, Al. Powstańców Wlkp. 72, 70-111, Szczecin, Poland

**Keywords:** Selenium, Colorectal, Cancer risk, Dietary supplement

## Abstract

**Background:**

Selenium has attracted attention because of its antioxidant properties. Antioxidants protects cells from damage. Certain breakdown products of selenium are believed to prevent tumor growth by enhancing the immune cell activity and suppressing the development of tumor blood vessels. In this observational study, selenium level was measured in a series of patients from Poland and Estonia to determine a correlation between levels of this microelement and colorectal cancer risk.

**Methods:**

A total of 169 colorectal cancer patients and 169 healthy controls were enrolled in the study after obtaining their informed consent. Selenium level in the blood serum was measured using Graphite Furnace Atomic Absorption Spectrometry (GFAAS). The statistical analysis was performed by Fisher’s exact test.

**Results:**

The threshold point of selenium level was 55 μg/l and 65 μg/l for Poland and Estonia respectively, for an increase in cancer risk. The lower levels of selenium were associated with greater risk of colorectal cancer.

**Conclusions:**

The result reveals a significant strong association between low selenium level and the colorectal cancer risk in both Estonian and Polish populations.

## Background

The efficacy of selenium is related to a “U” shaped curve in terms of the health benefits. The low levels of selenium is associated with an increased risk of malignancy [1,2]. Too much selenium (selenosis) is also linked to higher occurrence of common diseases, such as type 2 diabetes [2] and has also been shown to be linked with increased incidence of lung malignancy [3].

Selenium occurs naturally in the environment but with different levels. For example, people of Central Europe have very low exposure to selenium compared to higher endogenous levels in North America [4]. Much emphasis has been placed on selenium supplementation such that in some geographical regions the average daily intake far exceeds the requirement. In others, where background selenium concentrations are low, supplementation should prove to be much more beneficial in terms of overall health benefits. Selenium supplementation will afford different effects across large geographical areas and potentially adds to confusion about the beneficial effects of selenium supplementation. Moreover, intake of Se varies considerably between countries and different regions owing largely to the variability of the Se content present in plant foods and food chain [4].

Early detection of colorectal cancer (CRC) represents the best approach in reducing the burden of this disease on society. Genetic studies have been extremely useful in identifying markers that are associated with inherited forms of the disease. But for cases without apparent familial history or inherited component, it remains a challenge to identify individuals at risk of disease.

It has been reported [5] that patients with fasting levels of selenium below 128 μg/l are much more likely to present with one or more adenomatous polyps (OR 4.2). The same characteristics has been confirmed in number of other studies [6,7]. However, other studies do not support a protective role for selenium [8] against the risk of colorectal cancer. The recent SELECT trial also indicated that supplementation with selenium did not provide any immediate benefit [9] and indeed could result in increased prostate cancer incidence [10]. The critical factor in the SELECT trial is the average selenium level for each of the participants. In North America the average reported selenium level ranged from 122.4 μg/l – 151.8 μg/l. In Eastern Europe the average selenium level is ~70 μg/l, which is likely to have significant consequences when manipulated by supplementation.

In this observational study, we investigated the selenium concentration in a series of colorectal cancer patients from Poland and Estonia (both countries with low selenium levels) to determine whether there was a correlation between low levels of this micronutrient and colorectal cancer.

## Materials and methods

### Material

A total of 169 colorectal cancer patients and 169 healthy controls were enrolled in the study after obtaining their informed consent. There were 67 patients with colorectal cancer from Polish population and 102 colorectal cancer patients from Estonia. The matched unaffected control for each case/patient included for the studies were registered at International Hereditary Cancer Center, Pomeranian Medical University of Szczecin, Poland and the Estonian Genome Centre, University of Tartu, Estonia. Participants were matched for year of birth (+/− 3 years) and gender. In addition, participants from the Polish population were also matched for any malignancies among 1st degree relatives.

The Table 1 shows the characteristics of the individuals included in the study.

**Table 1:** Characteristics of subjects enrolled in the study

**Polish group**	**Cases**	**Controls**
Year of birth - average	1944	1944
- range	1926 – 1973	1928 – 1971
First-degree relatives		
- with colorectal cancer	9	9
- another cancer site	26	26
**Estonian group**		
Year of birth - average	1937	1938
- range	1920-1966	1920-1965
First-degree relatives		
- with colorectal cancer	3	-
- another cancer site	-	-

### Methods

The study has been conducted in accordance with the Declaration of Helsinki and all participants signed an informed consent document prior to donating a blood sample. Furthermore, the study was approved by Ethics Committee of Pomeranian Medical University under the number KB-0012/73/10. The blood (~10 ml) was collected from each person enrolled in the study in order to secure the blood serum. Selenium level in blood serum was determined using Graphite Furnace Atomic Absorption Spectrometry (GFAAS) – Perkin Elmer. Seronorm™ – Nycomed Pharma AS, Oslo, Norway was used as standard to validate the measurement method. The measurement accuracy was +/− 5% μg Se/l. Statistical analysis included the comparison of the Se level [μg/l] in each groups. Odds ratios were generated from two-by-two tables and statistical significance was assessed using the Fisher’s exact test. Odds ratios were calculated for sliding windows at constant amount of selenium concentration data points to determine the relationship between selenium concentration and disease risk. The calculations and the graphics were performed using a free statistical software package of R version-2.13.1.

## Results and discussion

**Figure 1:**
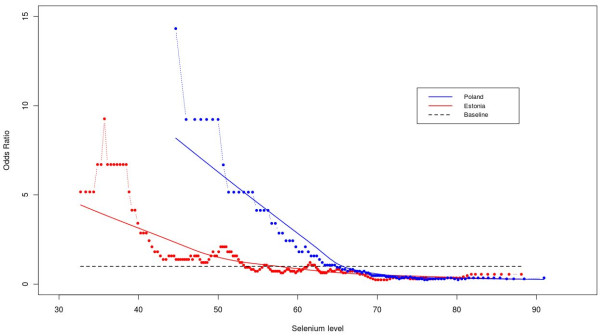
**Selenium level and colorectal cancer risk.** The relationship between selenium concentration in blood serum and the risk of colorectal cancer is displayed in form of odds ratios at each of the selenium level, separately for Patients from Estonia (in red) and Poland (in blue). The odds ratios are calculated for sliding windows of 30 selenium values each. The multiple regression model "lowess" is represented as a continuous line separately for each country. The reference baseline odds ratio of 1 has been added as a dotted black line.

Selenium levels were assayed in both the control and colorectal cancer groups. Selenium concentration of blood serum was plotted against odd ratio for each group of patients from Estonia and Poland (see Figure 1). The threshold point of selenium concentration was 55 μg/l and 65 μg/l for Poland and Estonia respectively, for an increase in cancer risk. The lower levels of selenium were associated with greater risk of being diagnosed with colorectal cancer. Based on the results shown in Figure 1, we divided the entire set of 169 cases and 169 controls into four groups (Table 2). Colorectal cancer patients were significantly more likely to be in the lowest selenium group compared to all other groups. A gradient effect was observed such that the number of colorectal cancer was lower as selenium concentrations approached the highest level.

**Table 2:** Selenium concentration in the colorectal cancer group and the healthy control group

**Groups**	**Selenium concentration μg/l**	**No. of colorectal cancer cases**	**No. of controls**
I	<46	53	12
II	46-58	47	27
III	59-71	44	52
IV	>72	25	78

Selenium concentration divided into groups.

The four groups were compared to each other to further define the correlation between selenium level and occurrence of colorectal cancer (Table 3). There was a gradient response, which showed the greatest effect between the groups with the lowest selenium concentration compared to the highest with OR (13.78). The effect was observed with reducing differences in selenium concentration and disease risk (see Table 3).

**Table 3:** The odds ratios of having low selenium compared to high selenium and the risk of colorectal cancer

**Comparison groups**	**p**	**OR**	**95% CI**
I vs II	0.0231	2.537	1.16 - 5.56
I vs III	<0.0001	5.22	2.48 - 11.0
I vs IV	<0.0001	13.78	6.31 - 29.82
II vs III	0.0293	2.07	1.11 -3.83
II vs IV	<0.001	5.43	2.82 - 10.44
III vs IV	0.0012	2.64	1.44 - 4.83

The results supports the consistent evidence of inverse correlation of selenium with colorectal cancer risk in countries with low endogenous levels of selenium i.e. lower circulating selenium levels being associated with the greatest risk of malignancy. A similar pilot study, examining a larger cohort of patients with colorectal lesions (colorectal adenomas) that included 451 cases where 69% have Se concentrations <140 μg/l and 31% with >140 μg/l, suggested a reduced prevalence of colorectal adenomas at higher selenium levels [11]. The other studies published in 1977 on selenium dietary intakes and CRC are derived from Schrauzer and colleagues. In these studies comprising 27 countries evaluation of the content of selenium in the diet showed an inverse correlation between the amount of selenium intake and mortality caused by cancer, including cancer of the colon and rectum. In the same study similar inverse correlations were found between cancer mortalities and the selenium concentrations in whole blood collected from healthy human donors in the U.S. and different countries [6]. The most fundamental results on the role of selenium supplementation in cancer prevention, including prevention of CRC, have been achieved in NPC study (Nutritional Prevention of Cancer). This was a randomized clinical trial designed to evaluate the effect of selenium supplementation of 200 μg daily selenized yeast in cancer prevention among 1312 residents of the Eastern United States. In this study statistically significant decrease in the incidence of cancers has been demonstrated only for subgroup of subjects with baseline selenium concentration <105 μg/l. It was observed also that the effective prevention on selenium supplementation depends not only on its initial level in the body but also on the cancer site. Selenium supplementation was associated with decreased risk of prostate, colon and lung cancers but reverse effect of increased risk was observed for breast cancers [7]. The further study performed by these researchers demonstrated that selenium supplementation was associated with a significantly reduced risk of prevalent colorectal adenoma, but only among subjects with a low baseline selenium level (<105,5 ng/ml, OR-0,27, p= 0,01) [12]. The subsequent studies coming from U.S.A. has been reported that patients with fasting levels of selenium below 128 μg/l are much more likely to occur with one or more adenomatous polyps (OR 4.2) [5]. The other study conducted at University of North Carolina on a relatively small group of participants (37 cases, 36 non-cases) showed that lower plasma selenium levels were associated with occurrence of multiple adenomas but not with adenoma size or location [13]. These reports are in agreement with our findings, where we have found that higher levels of Se are associated with a reduction in colorectal cancer risk.

Many authors concluded, that observations from the SELECT study are in opposite to above findings. The SELECT trial indicated that supplementation with selenium did not provide any immediate benefit in cancers prevention including CRC. It was a large, phase 3 randomized, placebo-controlled trial involving 35 535 men from 427 participating sites in the United States, Canada, and Puerto Rico. Results of the SELECT study showed that selenium supplementation did not reduce prostate cancer incidence, as well as CRC [9]. However, the trial included almost no participants within the range of selenium status (<106 μg/l in plasma) assuring benefit from selenium supplementation on prostate cancer risk [7,12].

In men in SELECT study, the IQR of baseline serum selenium was 122·4–151·8 μg/l, which, according to the NPC trial, put them into the category of non-significant increased risk from selenium supplementation [2]. In our opinion, despite negative results it is incorrect to say that SELECT provides an evidence for the lack of effectiveness of selenium supplementation in cancer prevention. Conversely, results of SELECT confirm observations of NPC study that selenium supplementation strongly depends on baseline selenium concentration and is beneficial only for individuals with low selenium level (<106 μg/l). The men who participated in SELECT study had baseline selenium level ~136 μg/l, which is too high to provide any protective effect of selenium supplementation in this group [9]. Therefore results of SELECT, even though they are extremely important, do not explain the effect of selenium on men of low selenium status.

Several studies from the other countries have provided some evidence for a beneficial effect of selenium on CRC risk. In the study from the Canada performed on a total of 1048 incidence cases (402 CRC) a statistically significant inverse associations between toenail selenium level and the risk of colon cancer for both genders combined (OR, 0.42; 95% CI, 0.19-0.93; p = .009) and for female subjects (p = .050) were observed [14]. The results of the retrospective study performed in Germany showed, that patients with a selenium level <70 μg/l had a significantly lower mean survival time and a lower cumulative cancer-related survival rate than patients with a selenium level >70 μg/l (p = 0.0009) [15]. The association between selenium status and risk of large size colorectal adenomas in a geographical area with a low selenium status were described also by group from the Spain. In this study a significant inverse association between selenium status and the diagnosis of large size adenomatous polyps after adjusting for confounding variables was found (adj. p = 0.029). Subjects with higher selenium status (> or = 75th percentile value of 82.11 μg/l) had a lower probability to be in the adenoma group than subjects with lower selenium status (< 82.11 μg/l, OR = 0.17, 95% CI = 0.03-0.84) [16]. Another study performed on 8,113 persons examined in 1972 from two counties in Eastern Finland showed that serum selenium of less than 45 μg/l was associated with a relative risk of cancer of 3.1 (95% confidence interval, 1.5-6.7, p < 0.01), including CRC [17]. Generally, several studies from Asia also supported a protective association between selenium and colorectal cancer. An example can be derived from the study of Chinese and Japan centers. In the study performed by Zhao et al. significantly lower selenium values were observed in blood from colorectal cancer patients than from normal individuals [18]. Nomura et al. demonstrated an increase in relative risk for colon for subjects in the lowest quintile of selenium values, as compared to the RR for subjects in the highest quintile, (RR = 1.8) [19].

Also the previous small studies (19 stomach cancers, 25 CRC, 25 controls) performed in Poland in Upper Silesia have shown that plasma selenium concentrations in gastrointestinal cancer patients (37.0 +/− 11.05 ng Se/ml or 38.4 +/− 12.6 ng Se/ml in stomach or colon cancer patients, respectively) were significantly lower as compared to the healthy age-matched control group (51.4 +/− 14.4 ng Se/ml) [20].

Some reports provided data which are in opposite to a beneficial effect of selenium on CRC risk. In study performed by Takata et al. no protective effect of selenium on CRC was observed among postmenopausal women recruited from 40 clinical centers across U.S. [8]. Association between selenium levels and the risk of CRC was not observed also in the Dutch study [21]. One of Finnish study indicated that high selenium intake and possibly also high vitamin E intake, especially among men, may provide protection against cancer of the upper gastrointestinal track but not against CRC [22]. But the latest reports are exceptions.

Generally, according to reported findings, it can be concluded that one of the key factor affecting the protective effect of selenium against CRC is the baseline selenium concentration. The protective effect of supplementation with selenium is beneficial in patients with relatively low (<100 μg/l) baseline concentration of selenium. Selenium supplementation in individuals with its higher baseline level is not protective and can even have an opposite effect of increasing cancer risk. Such reasoning is consistent with the view represented in the publication by Rayman were it is presented by the author as the “U” shaped curve [2]. Accordingly, in countries such as Poland and Estonia the risk of CRC may be higher than in the U.S. Actually, such validation of reported correlation is needed, however at present it is not possible due to differences between countries in the intensity of surveillance.

Supplementation with Se at the recommended optimal dose is safe and has no effect. Higher than recommended dose leads to selenosis and hence patients should be informed about the maximal daily dosage of selenium.

The background levels of selenium have a profound effect on any expected response from supplementation. The response on supplementation will be very different in countries with low endogenous levels of selenium in comparison to the higher levels.

Genetic variation in the proteins that utilize Se may affect its biological availability and population specific differences in genetic variation may also contribute to the responses observed [23]. Our result show greater risk of colorectal cancer in countries with low endogenous levels of selenium. Since there is also an association with higher levels of Se, any supplementation should be monitored to ensure that optimal levels are being attained and maintained. It remains to be seen if Se supplementation will alter colorectal cancer in countries where endogenous Se is low.

The average Se concentration in Poland and Estonia is ~ 80μg/l and colorectal cancer appears to be significantly more frequent in persons with low Se levels. Hence, it is not unreasonable to suggest selenium concentration as a surrogate marker to stratify persons for colonoscopy screening. Furthermore, it seems that in countries where selenium levels are low (~ 75–80 μg/l) consideration should be given to preventive measures that should include: a) screening persons for low levels of selenium; b) offer bowel cancer screening (colonoscopy, sigmoidoscopy or faecal occult blood testing); c) increase (or decrease) selenium to optimal levels through the use of dietary supplements, d) regular (i.e. every 12 months) measurement of selenium levels.

## Conclusions

The study shows a very strong correlation between the level of selenium in serum and the risk of colorectal cancer in both Estonian and Polish populations. Due to the risk of colorectal cancer, the majority of people from the Estonian and Polish populations should be advised to increase their selenium levels to approximately 100 μg/l.

The Prospective studies will be required to elucidate: a) possibility of dietary supplementation of selenium; b) the use of selenium measurements as markers of risk of colorectal cancers. Actually, assessment of selenium levels may increase the effectiveness of many screening programs for colorectal cancer, for example, the cost of detecting asymptomatic colorectal cancer by colonoscopy can be potentially reduced by targeting patients with low selenium.

## Competing interests

The authors declare that they have no competing interests.

## Authors’ contributions

Conceived and designed the experiments: MRL, RJS, AJ, AM, JL. Performed the experiments: MRL, SG, MT, MK, AV, KJ-B, KD, MM, GS. Analyzed the data: MRL, RJS, SG, MT, AM, MLT, PSF, JK, JL. Contributed reagents/materials/analysis tools: MRL, SG, MK, AV, KJ-B, KD, MM, GS, JL. Wrote the paper: MRL, SG, RJS, MT, AM, JL. All authors read and approved the final manuscript.

## Pre-publication history

The pre-publication history for this paper can be accessed here:

http://www.biomedcentral.com/1471-2407/13/214/prepub
